# Status, challenges, and prospects of artificial intelligence application in gout diagnosis and treatment, drug research and development, and disease monitoring

**DOI:** 10.3389/fmed.2026.1818451

**Published:** 2026-07-21

**Authors:** Jing Ma, Jing Zhao, Xinru Liu, Jin Yan, Yunxia Hou, Chunlei Li, Dafu Man, Cheng Wang, Hongbin Li, Yong Wang

**Affiliations:** Department of Rheumatology and Immunology, Inner Mongolia Key Laboratory for Pathogenesis and Diagnosis of Rheumatic and Autoimmune Diseases, The Affiliated Hospital of Inner Mongolia Medical University, Hohhot, China

**Keywords:** application status, artificial intelligence, challenges and prospects, diagnosis and treatment, gout

## Abstract

Gout is a chronic inflammatory disease caused by the deposition of monosodium urate crystals. Its global prevalence is rising, with an observable trend toward an earlier age at onset. Clinical management faces several persistent challenges, including delayed diagnosis, inaccurate disease assessment, a lack of individualized treatment strategies, challenges in long-term follow-up, and suboptimal patient adherence. In recent years, artificial intelligence (AI) has demonstrated significant potential across the gout care continuum. Leveraging its robust capabilities in data processing, pattern recognition, and predictive analytics, AI offers novel approaches to address these clinical challenges. This review systematically collates published investigations concerning AI in the diagnosis and clinical management of gout, delineates the current developmental stage of relevant work, and outlines viable directions for subsequent research in this domain.

## 1 Introduction

Gout, as a common inflammatory arthritis (1), has shown a significant upward trend in global incidence in recent years (2). In 2020, 55.8 million people globally had gout, with an age-standardized prevalence of 659.3 per 100,000, an increase of 22.5% since 1990. Age-standardized gout prevalence in 2050 is forecast to be 667 per 100,000 population. In 2020, the age-standardized prevalence of gout was highest in the regions of high-income North America, Australasia, and southern Latin America. By contrast, Tropical Latin America, the Caribbean, and central Latin America showed the lowest age standardized prevalence (3). Gout’s pathogenesis is primarily driven by hyperuricemia, which leads to the deposition of monosodium urate crystals in joints and surrounding tissues, triggering acute inflammation, chronic joint damage, and tophus formation (4). Currently, the continuous growth of gout patients has become an important public health issue: from the patient’s perspective, recurrent episodes of the disease can cause joint dysfunction or even disability, seriously restricting their daily life and work and greatly reducing their quality of life; from the medical and health perspective, the diagnosis and treatment needs of a large number of patients not only occupy limited specialized medical resources, but also further increase the economic burden on the medical and health system due to the subsequent management of complications (5).

Traditional gout diagnosis mainly relies on clinical symptoms, laboratory tests, and imaging methods (6). However, these methods have certain limitations. Objective data show that gout may sometimes present clinical features similar to other arthritis, including rheumatoid arthritis, psoriatic arthritis, and degenerative diseases (7), spondylarthritis (8) and other crystal arthropathies (such as calcium pyrophosphate deposition disease (CPPD) (3), making diagnosis difficult. In addition, blood uric acid levels fluctuate. Under certain conditions, individuals with normal blood uric acid levels may also experience gouty arthritis (9); thus, relying solely on uric acid levels may lead to missed diagnosis. Imaging examinations are one of the important methods for gout diagnosis, but relevant studies have shown that dual-energy computed tomography (DECT) has limited sensitivity in diagnosing acute gout patients, especially those with the first attack (10). Therefore, there is an urgent clinical need to develop more accurate and efficient gout diagnosis and treatment strategies. Against this background, artificial intelligence (AI) technology, by virtue of its advantages in data processing and pattern recognition, provides a new approach to address these clinical challenges. This review systematically summarizes the application status, existing challenges, and future prospects of AI in gout diagnosis and treatment, drug development, and disease monitoring.

## 2 AI overview and healthcare applications

AI is a branch of computer science that uses algorithms and statistical models to perform tasks that traditionally require human intelligence, such as perception, reasoning, learning, and decision-making (11). This technology is based on statistical data analysis or machine learning principles and can be divided into subfields, including machine learning, deep learning, computer vision (12), and Natural language processing (NLP). Machine learning excels at interpreting massive datasets and accurately evaluating complex patterns (13). It uses computation to simulate human pattern-recognition ability, enabling computers to improve their performance through data learning without explicit programming instructions (14). In gout-related research, we can collect a large amount of clinical data from gout patients, including clinical manifestations, laboratory indicators, and imaging features (15), to build machine learning models. This helps achieve gout diagnosis, disease assessment, and treatment plan prediction. Deep learning, a subset of machine learning, has emerged as its predominant methodology (16, 17). It has achieved remarkable success in image recognition, speech recognition, and natural language understanding (18). Deep learning methods can improve the efficiency of certain clinical tasks and provide powerful technologies for generating new knowledge and insights. They are especially useful for analyzing unstructured data such as text and images—tasks that were previously difficult to accomplish (17). Computer vision can directly recognize objects from raw image pixels. Specialists first label each image with the correct diagnostic result, known as the “*ground truth*.” Then, computer vision focuses on image and video recognition, handling specified tasks such as object classification, detection, and interpretation to classify predefined results (19). NLP, another subfield of AI, focuses on understanding human language. Researchers can use NLP analysis engines to identify common terms and concepts in social media data (20). For example, one study achieved 82% sensitivity and 92% specificity in identifying gout flares by combining NLP with machine learning techniques applied to unstructured electronic health records (21). As an emerging field, AI has been widely used in medicine and is regarded as the future of biomedical research, personalized medicine, and computer-aided diagnosis (22).

## 3 AI mining of medical data in gout

Gout flares are key outcome measures in gout treatment research. However, their transient and recurrent characteristics make it challenging to accurately identify them in large-scale databases, primarily due to the limited specificity of standard diagnostic codes (23). NLP, a subfield of AI, can address challenges in medical text processing—such as clinical note analysis and entity recognition. It acts as a pivotal technology linking unstructured electronic health record (EHR) data to clinical decision-making (24) and serves as an ideal technical solution to overcome these difficulties. First, NLP can screen and analyze massive datasets to identify gout-related disease patterns, symptoms, and risk factors, thereby facilitating early disease detection and diagnosis (25). Researchers have combined NLP with machine learning to develop gout flare detection algorithms. These algorithms not only retrospectively identify gout episodes from medical records but also successfully identify risk factors for gout flares (26). Second, advanced NLP models can effectively answer patient inquiries about their condition and treatment, thus improving the quality of patient health education (27). Furthermore, NLP technology supports the development of personalized treatment plans and facilitates sophisticated disease management, providing an alternative to the traditional one-size-fits-all treatment model (24).

A limitation of traditional NLP, however, is its potential underperformance when handling polysemous terms (words with multiple meanings). This has spurred the adoption of large language models (LLMs). Studies show that compared with traditional NLP techniques, LLMs can accurately identify disease diagnoses in electronic health records, even when disease names are highly similar to other vocabulary terms. They also exhibit excellent performance in linguistic contexts outside their core training datasets (28). These findings pave the way for cost-efficient disease detection from the extensive clinical electronic medical records of hospitals. Consequently, LLM technology holds promise for supporting diverse clinical applications, including the establishment of multilingual disease registries, the optimization of care assessments, and the improvement of patient recruitment efficiency for clinical trials (28).

## 4 Application of AI in gout diagnosis

### 4.1 Diagnostic models based on clinical data

Machine learning is a robust set of algorithms (29) that has also demonstrated significant application value in the clinical diagnosis of gout. Researchers have employed machine learning methods to construct predictive models using clinical data from gout patients. These models are trained to identify and analyze patterns within the data, facilitating a thorough investigation of the factors influencing tophus formation. This helps develop more appropriate clinical research protocols for gout patients with tophi, refine the diagnostic system for tophi, and provide clinical evidence for their early treatment (29). Similarly, machine learning plays an important role in optimizing gout diagnostic techniques. Studies have applied machine learning to the complex latent Raman spectra of clinical patient samples collected at primary care settings. Notably, such favorable diagnostic performance was only validated using samples from a single clinical center in existing research, which restricts the generalizability of these Raman spectrum-based algorithms; independent external validation datasets from multiple hospitals and geographic regions are still necessary before this detection technology can obtain FDA or IVDR certification for routine clinical deployment (30). It thus provides a convenient and efficient solution for accurate diagnosis in primary healthcare institutions and resource-limited settings. Furthermore, building machine learning models for gout prediction using clinical data and ultrasound features lays a foundation for future clinical decision support applications in gout diagnosis (22). In addition, studies have shown that gout and hyperuricemia have distinct serum metabolomic profiles. AI diagnostic models may therefore improve current gout management by enabling early detection or prediction of hyperuricemia progression to clinical gout (31).

### 4.2 AI-powered automatic ultrasound image detection for assisted diagnosis

Ultrasound examinations have become a vital diagnostic tool for gout due to their non-invasive nature, good cost-effectiveness, and real-time imaging capabilities (32). AI technology demonstrates significant potential in ultrasound image analysis. Researchers have trained deep learning algorithms using extensive ultrasound datasets from gout patients to develop AI models capable of automatically detecting gout through ultrasound imaging. Furthermore, these models generate interpretable visual heatmaps to localize gout-related pathological features, providing visual support for clinical decision-making (32). Studies indicate that deep learning-trained AI systems can identify tophi in ultrasound images with high accuracy (33). Compared to traditional ultrasound interpretation, AI-assisted diagnosis not only offers an objective, standardized approach to gout diagnosis, reducing diagnostic variability among clinicians, but also provides real-time diagnostic recommendations. This helps clinicians quickly identify patients with early-stage gout and facilitates timely treatment decisions—which is particularly crucial during acute gout flares (22).

### 4.3 Combination of AI and DECT for improved diagnostic accuracy

Inflammatory reactions induced by monosodium urate crystal deposition can trigger gout flares (34). DECT is renowned for its ability to effectively distinguish monosodium urate crystals from other high-attenuation materials (35). It is recommended by the European League Against Rheumatism for the precise assessment of monosodium urate deposits, demonstrating superior detection capabilities compared to ultrasound examinations and clinical evaluations (34). The integration of AI technology with DECT further enhances diagnostic efficacy. Research indicates that deep learning reconstruction not only is expected to improve the detection sensitivity of monosodium urate crystals but also offers advantages in reducing image noise, enhancing image contrast and overall image quality, and enabling radiation dose reduction (36). For instance, leveraging high sensitivity, specificity, and accuracy, deep learning in DECT facilitates gout diagnosis, precisely quantifies crystal deposits, provides information for clinical decision-making, and optimizes patient care by promoting early diagnosis and timely intervention (35). Moreover, deep learning models can shorten radiologists’ image interpretation time while enhancing diagnostic accuracy (37) thus helping reduce radiologists’ workload (16) and enabling automated assessment of musculoskeletal pathologies (38).

### 4.4 Clinical practice of AI-assisted diagnosis and treatment system

The AI standardized diagnosis and treatment system combines parallel intelligence theory with the clinical expertise of top medical specialists. By constructing precise diagnostic and therapeutic models, it assists clinicians in standardizing treatment pathways and standardizing the therapeutic effects of hyperuricemia and gout (39). In practical clinical application, after physicians input patient-specific information, the AI-assisted diagnostic system rapidly generates diagnostic recommendations. These encompass potential diagnoses, key differential diagnosis considerations, and suggestions for further investigations, thereby supporting clinicians in making more scientifically grounded and rational diagnostic decisions (6). Taking the Parallel Gout Diagnosis and Treatment System deployed at Qingdao University Hospital’s gout clinic as an example, this system not only overcomes limitations in gout management arising from variations in physician expertise but also provides patients with round-the-clock personalized guidance, supporting chronic disease management and condition control. This represents the evolution of traditional healthcare models toward intelligent, parallelized approaches (40). Furthermore, research indicates that the use of AI-based intelligent gout management systems is associated with a reduced incidence of chronic kidney disease stage ≥3 in gout patients and an improved rate of target serum urate attainment. Consequently, such intelligent management systems represent a novel approach to enhancing the actual renal status of gout patients and increasing the success rate of urate-lowering therapy (41).

### 4.5 Application of AI in the differential diagnosis of gout

Studies have shown that both gout and CPPD can be objectively diagnosed with a machine learning algorithm acting on a large set of >20,000 Raman spectra from synovial fluid samples of 446 patients. The designed models reached an accuracy of 92.5% for gout and 96.0% for CPPD, which is comparable to the diagnostic accuracy of Raman spectroscopy when spectra are classified by a trained observer (30). Existing studies have also explored the application of NLP in rheumatology and found that it holds potential for detecting diseases and conditions from unstructured reports, especially for rheumatoid arthritis, spondyloarthritis, and gout. However, the realization of this potential is still in its early stages. To achieve the full benefits of NLP, it is necessary to overcome existing limitations through focused research, ethical commitment, and ongoing technological enhancements (27). In addition to traditional NLP techniques, LLMs are also being used to aid in differential diagnosis. One study proposes and tests a framework to detect disease diagnosis using a recent LLM, Meta’s Llama-3-8B, on EHR documents. The framework was developed using a training and testing set of 700 paragraphs assessing ‘gout’ from a random selection of EHR documents. The framework was further validated on 600 paragraphs assessing CPPD. The results demonstrated that LLMs can accurately detect disease diagnoses from EHRs. Limitations are also evident, including single-center design, single-language application, and exclusive focus on the identification of diagnostic results, which need to be further improved in future studies (28).

## 5 Transformative role of AI in gout drug development

Deep learning algorithms demonstrate significant potential in predicting drug efficacy and designing novel therapeutics (11). Through deep learning approaches, the elucidation of allosteric pathways and investigation of the specific mechanisms underlying the non-competitive inhibition of xanthine oxidase by two distinct peptide inhibitors provide novel insights into food-derived anti-xanthine oxidase inhibitors. This will aid in developing side-effect-free urate-lowering agents derived from food sources (42). Machine learning methods are similarly employed to screen natural compounds with inhibitory activity (43). Researchers developed quantitative structure–activity relationship models by integrating diverse machine learning approaches and three descriptor pools. These models revealed key characteristics of xanthine oxidase inhibitors, identifying vanillic acid as a promising novel xanthine oxidase inhibitor candidate with potential as an effective agent for gout management (44). The integration of AI and machine learning technologies with conventional research methodologies has been increasingly adopted for target identification in hyperuricemia, gouty arthritis, and hyperuricemic nephropathy (45). For example, by combining association rule learning with gene network analysis, a puuD gene-encoded integral membrane protein with a C-terminal cytochrome c was identified as the missing urate oxidase with a domain of unknown function (DUF989) responsible for the biochemical activity, completing the link in the conversion of urate into allantoin and pointing out an enzyme useful for drug development (46).

## 6 Intelligent monitoring and management of gout conditions

Wearable sensors integrating medical technology with AI analysis enable continuous remote monitoring, facilitating early disease detection and proactive intervention (47). Wearable electrochemical sensors have practical application potential in uric acid detection due to their simplicity of preparation, rapid testing, high sensitivity, and strong selectivity (48). Researchers used a visualized intelligent wearable sweat detection platform to collect surface-enhanced Raman spectroscopy data from human sweat containing uric acid. Subsequent analysis using AI algorithms demonstrated that artificial neural network algorithms within the AI framework can reliably identify gout with an accuracy of up to 97% (49). Beyond serum uric acid monitoring, fully integrated wearable sensor arrays can wirelessly and non-invasively measure various metabolites and electrolytes in raw sweat, saliva, or urine simultaneously when the subject is moving. This is achieved through seamless integration of specially designed microfluidic, sensing, and electronic modules. Furthermore, their practicality for non-invasive gout management in healthy subjects and gout patients was evaluated through purine-rich dietary challenges and drug treatment controls (50).

However, while elevated serum uric acid correlates with increased attack risk in gout patients, it does not perfectly predict subsequent episodes (51). Researchers are exploring the potential of machine learning in clinical prediction for diseases such as gout, which have complex metabolic and genetic backgrounds. For instance, integrating machine learning models with genetic information improves the accuracy of gout prediction in clinical practice (52). Moreover, machine learning analysis of the human gut microbiome enables predictive diagnosis of gout (53). Concurrently, machine learning predictive models developed from patient clinical data can assess the relationship between physical activity duration, sedentary time, and gout risk (54). Further research has identified multiple metabolites as biomarkers through rigorous variable screening and cross-validation of non-targeted metabolomics analysis, and has constructed predictive models using machine learning algorithms (55). These metabolites may serve as biomarkers for predicting gout flares, helping identify gout patients at high risk of flares (51). Computer-simulated quantitative biomarkers for hyperuricemia based on multimodal machine learning further enable personalized risk stratification for gout and metabolic outcomes. They reveal that lifestyle modifications can improve metabolic outcomes in high-risk populations, providing guidance for preventive interventions (13). Gout prediction models not only provide more accurate gout prediction results by analyzing massive clinical data and complex patterns, enabling early intervention and reducing gout prevalence, but also possess rapid learning and iterative capabilities. These models can be continuously updated and optimized using newly collected subject data (15), ensuring sustainability.

In addition to clinical data, AI can integrate other multi-source data, enabling comprehensive analysis of anthropometric data, lifestyle data (such as diet, exercise, and alcohol consumption), and nutritional characteristic data (56). Through the integration of multi-source data, we can more comprehensively and in-depth understand the disease risk and pathogenesis of patients, improving the accuracy and reliability of gout diagnosis. Studies have shown that risk prediction models trained using patients’ demographic characteristics, community and hospital diagnoses, regular drug prescriptions, and laboratory test results have relatively good performance and high predictive value in identifying hyperuricemia patients at risk of gout (57). Similarly, combining genetic data with clinical data to analyze the association between specific genetic variations and gout onset helps clarify the role of genetic factors in gout pathogenesis. This provides early warning and personalized diagnosis for patients with genetic susceptibility. Meanwhile, integrating lifestyle and nutritional characteristic data can evaluate the impact of these factors on gout onset, offering more targeted early intervention and treatment recommendations for patients (56).

## 7 Challenges and future perspectives

### 7.1 Data quality dependence and privacy security assurance

While integrating AI into healthcare offers numerous advantages, it also presents potential disadvantages and challenges that must be addressed (12). First, the accuracy and reliability of AI are highly dependent on the quality and quantity of training data. However, in the field of drug discovery, standardized datasets are often insufficient, making it difficult to effectively train AI algorithms using large datasets. Moreover, the efficacy of AI algorithms hinges on the quality and completeness of training data. If data is biased or incomplete, the resulting predictions and discoveries may be distorted or flawed (11). Furthermore, collected data may contain sensitive patient information, making compliance with relevant laws and regulations, alongside safeguarding AI systems against cyberattacks and data breaches, of paramount importance (58). Concurrently, AI models designed to assist patients in accessing information about rheumatic diseases may provide inaccurate information, thereby endangering patient health. Developers should source health-related educational data from reliable sources to mitigate such risks (59).

### 7.2 Dual challenges of ethical dilemmas and technical bottlenecks

Ethical issues are paramount and may significantly impact the application of AI as a guide for personalized drug therapy—particularly concerning drug selection and optimal dosage (60). From an ethical standpoint, developers bear the responsibility to ensure AI systems are rigorously designed and tested to prevent harm. This entails continuous monitoring and updating of AI models to guard against outdated or inaccurate predictions (58). Data ownership presents another ethical challenge, particularly in AI applications for drug development, which typically require patient consent for data usage. Such processes may spark conflicts among researchers, patients, and pharmaceutical companies regarding data ownership and the implementation of AI tools (47). Additional ethical concerns surround AI implementation: threats to data security, altered dynamics in doctor–patient relationships, potential exacerbation of social inequalities, and the development of AI-powered robots that may ultimately displace numerous professional roles, contributing to rising unemployment rates (61). Technical resource dependencies further compound the complexity of ethical implementation. Deep learning-based algorithms not only require vast datasets to outperform alternative methods but also rely on costly GPUs and hundreds of workstations (24). Despite these challenges, the future of AI in drug discovery remains promising. With advances in hardware, software, and AI algorithms (62), this technology is expected to play a vital role in gout diagnosis and treatment modalities.

### 7.3 Model interpretability

AI is rapidly permeating nearly all scientific domains (63). Integrating AI technology into digital healthcare systems will propel digital medicine into a new phase, particularly aiding the management of patients with rheumatic diseases (59). However, one of the most criticized characteristics of AI algorithms—their so-called black-box nature—undoubtedly poses challenges for the medical field. These algorithms are considered inherently non-interpretable (63), meaning that for many healthcare professionals, AI terminology and operational mechanisms remain akin to a black box. This duality fosters both inflated expectations and unfounded apprehensions (64). Under these circumstances, the field of explainable AI—AI models that are both interpretable and designed to enhance user comprehension and transparency, thereby boosting confidence and trust—has attracted growing attention. However, no authors have yet employed evaluation metrics specifically tailored for explainability, making the development of dedicated assessment methods for explainable AI an urgent research priority (65).

This schematic diagram demonstrates the applications of artificial intelligence in gout research and clinical practice. Through integrating, screening, and analyzing data, artificial intelligence directly or indirectly participates in multiple links during the diagnosis and treatment process of gout. AI/artificial intelligence, DECT/dual-energy computed tomography.

## 8 Conclusion

**Figure 1:**
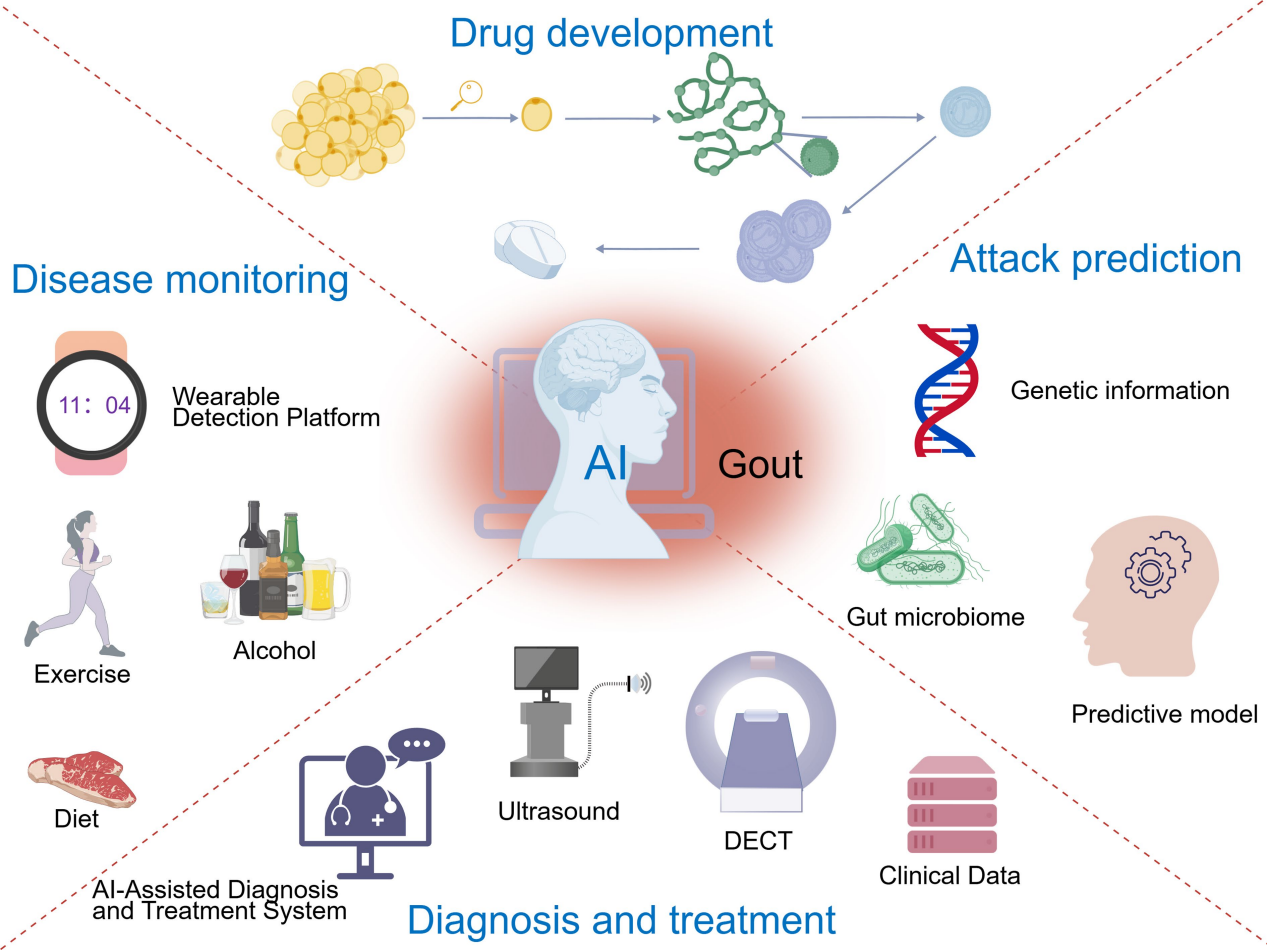
AI in gout, including diagnosis, treatment, drug development, attack prediction, and disease monitoring (created with BioGDP.com).

In summary, research regarding AI involved in the diagnosis and treatment of gout has covered a wide range of aspects (Figure 1). However, most investigations remain at an exploratory stage, and their findings require repeated verification through large-scale multicenter clinical trials. Representative cases across machine learning, deep learning, NLP, and computer vision are summarized in Table 1. Current studies demonstrate that, regarding medical imaging analysis, AI shows potential for precise identification of joint ultrasound images in retrospective research datasets, facilitating the identification of gout patients. Concurrently, it has the potential to optimize analytical workflows in DECT imaging diagnostics, which may improve diagnostic performance in limited research settings. At the data mining and analysis level, predictive and diagnostic models built upon clinical data, combined with multi-source data fusion analysis techniques, can integrate patient history, laboratory indicators, lifestyle factors, and nutritional characteristics to provide more comprehensive decision support for gout diagnosis. In addition, in the field of intelligent symptom recognition and diagnostic decision support, AI can assist in identifying disease diagnoses under experimental conditions but still lacks sufficient prospective clinical validation; it also assists in the establishment of standardized databases and the execution of rigorous disease assessment operations in research scenarios. The application of AI-assisted systems in clinical practice may help clinicians achieve relatively standardized diagnostic and therapeutic procedures in pilot settings, while large-scale real-world validation, physician comparative evaluation, and clinical outcome verification remain insufficient. Research on AI for the differential diagnosis between gout and other diseases remains relatively scarce, and there are substantial gaps regarding its implementation across diverse clinical scenarios.

**Table 1:** Evidence table of included studies.

Author	Year	Country	Sample size	Data type	AI method	Task type	Reference standard	Validation strategy	Main performance metrics	Whether external validation was performed	Key limitations	Level of clinical translation	PubMed link
Tang et al.	2025	China, Japan, and Republic of Korea	233 stool samples	16S rRNA gut microbiome sequencing data	Five machine learning algorithms: Adaptive Boosting (AdaBoost), Decision Tree (DT), Random Forest (RF), Support Vector Machine (SVM), and eXtreme Gradient Boosting (XGBoost); SHAP interpretability analysis was used to optimize model features.	Perform multi-class classification for healthy controls, hyperuricemia, and gout based on gut microbiota 16S rRNA sequencing data, and screen core microbial biomarkers via LEfSe and SHAP analysis.	The clinical diagnostic criteria of original published studies were used to strictly distinguish healthy controls, hyperuricemia, and gout subjects; OTUs were clustered at 97% sequence similarity, the SILVA 138.1 database was used for taxonomic annotation, and *p* < 0.05 was defined as statistically significant.	The dataset was split into training and test sets at an 8:2 ratio, and the training set was further divided into internal training and validation subsets at 8:2. Grid search was used for hyperparameter optimization, and 5-fold cross-validation was performed for the model.	Model fitting and parameter optimization outcomes were assessed using four evaluation metrics: accuracy, precision, sensitivity, and *F*1-score.	No	1. Samples were only collected from three Asian countries with limited cohort size and population diversity. 2. An independent external validation cohort is absent to verify the generalizability and robustness of machine learning models. 3. Reliance solely on 16S rRNA data fails to fully infer genetic information and actual functions of bacterial communities.	Basic research stage with no clinical application.	https://pubmed.ncbi.nlm.nih.gov/40640723/
Schmolke et al.	2025	Not explicitly stated in the main text	A raster phantom and an ex vivo porcine foreleg bio-phantom were constructed, containing syringes with four gradient-concentration MSU solutions and negative controls to simulate the anatomical environment of gouty tophi	Raw 320-row volume DECT scanning data of phantoms acquired at different tube currents, image data processed by four reconstruction techniques, as well as quantitative MSU volume detection data and image quality assessment data including signal-to-noise ratio (SNR) and contrast-to-noise ratio (CNR)	Two deep learning reconstruction (DLR) techniques (AiCE mild; AiCE strong) and two established methods—iterative reconstruction (IR) and filtered back projection (FBP) were compared.	To evaluate and compare MSU detection performance, detected volume, image quality, and dose-dependent characteristics between two DLR techniques and two conventional reconstruction methods.	Not explicitly stated in the main text	Not explicitly stated in the main text	1. Detection efficacy: Both DLR techniques outperformed FBP and IR in MSU volume detection at all concentrations in two phantoms; 2. Image quality: DLR reduced image noise and improved SNR, CNR, and overall image quality; 3. All four reconstruction methods exhibited positive correlation between detected MSU volume and CTDIvol in the raster phantom, while no such correlation was found for two DLR techniques in the bio-phantom; 4. Dose plateau effect: MSU detection capacity of all techniques reached a plateau at high radiation dose levels.	No	1. The results based on the rotate-rotate method have limited applicability to other DECT scanners and techniques; 2. Only a restricted range of MSU concentrations was analyzed; 3. DLR techniques from different vendors were not compared, limiting the generalizability of findings; 4. 60 keV virtual monochromatic images were used as surrogates for standard 80 kVp images with inherent limitations; 5. Reconstructed data were incompatible with some post-processing software versions; 6. No definitive reference standard for volume metrics was available; 7. No similarity metrics were included in evaluation, and all findings were obtained from phantom experiments without clinical validation.	This is an explorative phantom study. DLR improves MSU detection sensitivity, reduces image noise and radiation exposure, and shows potential for low-dose DECT gout diagnosis. Clinical validation in patients is required before routine clinical use.	https://pubmed.ncbi.nlm.nih.gov/40436916/
Castro-Zunti et al.	2025	Republic of Korea	Clinical DECT imaging data of 47 gout patients and 27 control subjects were included	Clinical DECT scan frame images with cropped green region-of-interest images containing true tophi and artifact interference data.	A three-stage machine vision pipeline was developed. The algorithm crops and filters green ROIs firstly. Three fine-tuned deep learning models were applied for classification of small, medium, and large ROIs based on pixel area statistics. A machine learning system finally realized patient-level classification with integrated ROI features.	To localize MSU crystal deposits on DECT images, distinguish gouty tophi from artifacts, and conduct patient-level gout classification to reduce false-positive diagnoses.	Gout patients scored ≥ 8 based on ACR/EULAR criteria with foot or ankle pain. Controls had no gout history, ACR/EULAR score < 8, no gout-related diagnosis codes, and no gout symptoms. Patients under 16 years old, patients with incomplete clinical records, and those with metal prosthesis were excluded.	6-fold cross-validation	Pipeline performance including accuracy, sensitivity, specificity and ROC AUC at both ROI level and patient level.	No	Limited training data size and diversity; no formal ROI annotation and ground truth bounding boxes for rigorous localization evaluation; VGG16 GAP was prioritized over more accurate ViTb16 for faster inference; only internal retrospective validation was performed, lacking multi-center, prospective and blinded external validation to verify generalizability and clinical efficacy.	It is the first automated DECT system for differentiating gouty tophi from artifacts. The system serves only as a diagnostic auxiliary tool, and final clinical diagnosis must be made by trained experts. Clinical testing and functional front-end development are required before clinical application.	https://pubmed.ncbi.nlm.nih.gov/41057720/
Niessink et al	2025	Netherlands	A total of 446 synovial fluid samples from patients with various rheumatic diseases were included, of which 246 were used for model training and 200 for model validation.	Raman spectra data of synovial fluid samples.	Two one-against-all classifiers were built in Python for MSU and CPPD, respectively. Disjoint principal component analysis (PCA) was applied to reduce spectral data into 10 key dimensions. Support vector machine (SVM) with a radial basis function kernel was used for crystal classification. t-SNE was utilized for data cluster trend analysis.	Construct machine learning models based on Raman spectra to achieve objective diagnosis of gout and CPPD, and classification of crystal types.	The primary reference was crystal identification results via polarized light microscopy (PLM) assessed by trained rheumatologists. The 2015 ACR/EULAR gout criteria and 2023 ACR/EULAR CPPD criteria incorporating laboratory, age, and radiological data were also applied for comprehensive judgment.	The dataset was split chronologically by patient presentation order. The first 246 samples were used for model training, and subsequent consecutive, unselected 200 new patient samples were used for internal validation.	Diagnostic accuracy measures including sensitivity, specificity, positive predictive value, negative predictive value, positive likelihood ratio, negative likelihood ratio, and accuracy with corresponding 95% confidence intervals were calculated using 2 × 2 contingency tables. Diagnostic performance was assessed for gout, CPPD, and combined test results.	No	1. Failed to identify rare pathogenic crystals such as BCP. 2. Only the PCA-SVM algorithm was used without comparison of multiple classification models. 3. Validation was limited to a single center without multi-center external validation.	Pre-clinical translational stage. It enables rapid and objective point-of-care crystal detection with clinical application potential, without large-scale clinical implementation.	https://pubmed.ncbi.nlm.nih.gov/39222431/
Faghani et al.	2024	United States	A total of 30 DECT examinations from different patients were included.	Dual-energy CT (DECT) imaging data from patients with and without gout, including material-decomposed 3-color pixel images (green for MSU deposits, blue for calcium, black for background) in original DICOM format, transformed to NIfTI format after preprocessing.	A UNet-based deep learning segmentation model that generates red masks to enhance the visibility of green foci of MSU deposits for automated detection. The model achieves 98.72% sensitivity and 99.98% specificity, with DSC of 0.9999 for background pixels, 0.7868 for green pixels, and average DSC of 0.8934 for both pixel types. It can generate a file containing coordinates of identified lesions to assist reading navigation.	Develop a UNet-based deep learning algorithm for automated detection of MSU crystal deposits on DECT images, explore its impact on radiologists’ reading time and gout diagnostic confidence, evaluate tool helpfulness, and calculate readers’ diagnostic accuracy, sensitivity, and specificity with and without DL assistance.	Gout cases were diagnosed according to the 2015 ACR/EULAR gout classification criteria, incorporating imaging findings, joint aspiration, serum uric acid level, and clinical evaluation.	Prospective, randomized, crossover study design. 30 DECT images were reviewed twice independently, with and without DL tool assistance, respectively, with a 2-week washout period between the two sessions. Half of the cases were randomly selected for tool-assisted review in each session, independently completed by an attending radiologist and an in-training fellow	1. Model performance: 98.72% sensitivity and 99.98% specificity for MSU deposit detection, with DSC of 0.9999 for background pixels, 0.7868 for green pixels, and average DSC of 0.8934 for both pixel types; 2. Evaluation indicators: Recorded radiologists’ reading time, binary gout diagnosis results, 5-point Likert-scale diagnostic confidence scores, and 3-point Likert-scale DL tool helpfulness scores; 3. Diagnostic efficacy: Calculated the diagnostic accuracy, sensitivity, and specificity of each reader with and without DL tool assistance;	No	1. Small number of reviewed cases and evaluation by only two readers; 2. The DL algorithm was trained on single-institution data without external validation, and the findings need further verification via larger-sample and multi-reader studies.	The DL algorithm can identify tiny MSU deposits to correct diagnosis and improve the accuracy of DECT-based gout diagnosis, and reduce reading time for inexperienced radiologists, showing promising clinical potential. Further algorithm optimization and large-sample studies are required to confirm findings and clarify long-term clinical value.	https://pubmed.ncbi.nlm.nih.gov/38440382/
Bürgisser et al.	2024	Switzerland	A total of 700 paragraphs assessing gout were used as the training and testing dataset, and additional 600 paragraphs assessing CPPD were adopted for further framework validation.	Unstructured French-language EHR documents, including sentences related to gout and CPPD.	Large language model (LLM), meta’s Llama- 3- 8B	Propose and test an LLM framework to detect disease diagnosis in unstructured French EHR documents. Evaluate framework effectiveness using the polysemous French term for gout, distinguish positive gout diagnosis from negative diagnosis, family disease history and other word usages, and validate the framework on a CPPD-related sentence dataset.	Two healthcare professionals (one internal medicine physician, one registered nurse) trained by a board-certified rheumatologist evaluated phrases to distinguish confirmed personal gout or CPPD diagnosis from negated diagnosis, family diagnosis, and alternate word usages. Disagreements were adjudicated by a board-certified rheumatologist to form the gold standard.	Further validated the detection framework using 600 CPPD text paragraphs	Algorithm performance, Sensitivity analysis	No	1. The method was only tested in a single academic institution; 2. The study only adopted French data, and results may vary in low-resource languages; 3. The model only identifies disease diagnosis without specific disease subtype classification; 4. The model cannot fully assess all elements of ACR/EULAR classification criteria; 5. Long dataset processing time limits frequent updates and rapid application scenarios.	Only model validation was completed. Further verification and optimization are required, and real-time clinical application has not been implemented.	https://pubmed.ncbi.nlm.nih.gov/39794274/

AI also exhibits promising research potential in gout condition monitoring and drug development. For condition monitoring, wearable devices integrated with AI technology enable non-invasive, real-time surveillance in experimental and small-sample research scenarios to aid gout identification while providing actionable data support for disease management. In drug development, AI shows preliminary potential to alleviate partial limitations of traditional research and development in preclinical studies: virtual screening technology can rapidly narrow the candidate scope and reduce the amount of experimental validation; deep learning can reveal drug action pathways and mechanisms, providing insights for the development of novel inhibitors; machine learning can construct quantitative models to screen natural active compounds, aiding in the discovery of potential urate-lowering agents, and could potentially optimize research and development efficiency while reducing costs at the computational research stage. Notably, these advantages are currently limited to preclinical and retrospective data analysis, lacking prospective clinical verification, real-world deployment assessment, and cost–benefit evaluation.

Despite its preliminary encouraging research outcomes, the application of AI in gout diagnosis and treatment faces multiple challenges requiring urgent resolution. At the data level, AI model training relies on vast quantities of high-quality, standardized, and representative data. Limited data availability hinders model development; while protecting sensitive patient information, ensuring data security, and preventing the dissemination of misinformation all require robust compliance management and reliable data sources. Ethically and resource-wise, developers bear the responsibility for the ethical design and rigorous validation testing of AI systems, necessitating continuous monitoring and updates to prevent inaccurate outcomes. They must also address ethical concerns such as data security, evolving doctor–patient relationships, potential exacerbation of social inequalities, and potential job displacement. Technologically, reliance on costly hardware and computational power further complicates ethical implementation. Additionally, insufficient model interpretability remains a critical bottleneck requiring innovative solutions.

Beyond the aforementioned issues, relevant research also presents multiple inherent limitations related to the clinical characteristics of gout. First, accurate labeling of monosodium urate (MSU) crystals remains challenging, which may affect the training quality and generalization performance of AI models. In early gout cases, dual-energy CT (DECT) is prone to artifacts and false-negative results, limiting the reliability of AI-based DECT image analysis. In addition, ultrasonic assessment of gout is highly operator-dependent, which introduces subjective variability and hinders the standardized application of ultrasound-based AI models in clinical practice. In terms of clinical data acquisition, electronic health records often contain incomplete documentation of gout flares, while serum uric acid levels are not completely consistent with the risk of acute flares, both of which may cause data bias in model construction. Furthermore, existing AI studies have rarely considered the potential model bias caused by differences in ethnic backgrounds, dietary patterns, and renal function status of patients. Meanwhile, current research lacks sufficient exploration on AI-assisted prediction of urate-lowering therapy adherence and the influence of comorbid chronic kidney disease on gout diagnosis and prognosis. In addition, long-term outcome data supporting the clinical application of AI models for gout are still insufficient. Currently, most existing AI models lack prospective multicenter validation, real-world clinical deployment assessment, physician head-to-head comparative trials, long-term clinical outcome verification, and cost-effectiveness analysis, which restricts their large-scale clinical translation. Furthermore, as technology advances and matures, these challenges are expected to be gradually resolved, enabling AI to serve as a promising auxiliary tool for the comprehensive improvement of gout diagnosis, treatment, and long-term management outcomes.
