# Supplementary material for: Status, challenges, and prospects of artificial intelligence application in gout diagnosis and treatment, drug research and development, and disease monitoring
Source: Front Med (Lausanne). 2026 Jul 21;13:1818451. doi: 10.3389/fmed.2026.1818451 (PMC13433292; doi:10.3389/fmed.2026.1818451)
Supplement: Supplementary file 1 [file Data_Sheet_1.PDF]

Generative Artificial Intelligence Usage Statement

Item	Details
AI Tool Name	DouBao
Model & Version	DouBao Large Language Model, Version: DouBao-202512
Developer/Source	ByteDance, https://www.doubao.com
Dates of Use	December 2–11, 2025
Purpose of Use	Translation, language polishing, grammar checking, and optimization of academic expression
nitial Prompts	• Prompt 1: "Translate based on the original Chinese text without altering its meaning. Reframe the content in an English context, avoiding obscure, rare, or archaic vocabulary. Ensure sentences are coherent and semantically smooth. Avoid complex sentences. Maintain seamless transitions and logical flow: [Paste Text]"<br>• Prompt 2: "Provide one-to-one translation without expanding the meaning. Ensure expressions align with English conventions. Use medical terminology appropriate for SCI journal publication. Write in American English: [Paste text]"
Final Prompts	• Prompt 3 (Final): "1. Evaluate whether the text meets SCI journal publication standards and whether it contains any fundamental errors. 2. If non-compliant, compare with the original Chinese text to ensure faithful preservation of meaning while performing polishing revisions. Bold any modified sections. Rewrite using English context appropriate for SCI journal publication, maintaining professionalism with medical terminology. Avoid obscure, rare, or esoteric vocabulary. Ensure sentence coherence and logical flow. Avoid complex sentences. Prioritize smooth transitions and logical consistency: [Paste Text]"
Verification Statement	All content generated or assisted by DouBao has been reviewed word by word by all authors to verify factual accuracy, scientific integrity, and originality. The authors confirm that the manuscript contains no plagiarized or unverified content and assume full responsibility for the final manuscript.
